# Sexual differentiation of the zebra finch song system: potential roles for sex chromosome genes

**DOI:** 10.1186/1471-2202-10-24

**Published:** 2009-03-23

**Authors:** Michelle L Tomaszycki, Camilla Peabody, Kirstin Replogle, David F Clayton, Robert J Tempelman, Juli Wade

**Affiliations:** 1 Department of Psychology & Program in Neuroscience, Michigan State University, East Lansing, MI, USA; 2 Department of Zoology, Michigan State University, East Lansing, MI, USA; 3 Department of Cell and Developmental Biology, Institute for Genomic Biology, Neuroscience Program, University of Illinois, Urbana-Champaign, IL, USA; 4 Department of Animal Science, Michigan State University, East Lansing, MI, USA; 5 5057 Woodward Ave, Suite 7908.1, Department of Psychology, Wayne State University, Detroit, MI 48202

## Abstract

**Background:**

Recent evidence suggests that some sex differences in brain and behavior might result from direct genetic effects, and not solely the result of the organizational effects of steroid hormones. The present study examined the potential role for sex-biased gene expression during development of sexually dimorphic singing behavior and associated song nuclei in juvenile zebra finches.

**Results:**

A microarray screen revealed more than 2400 putative genes (with a false discovery rate less than 0.05) exhibiting sex differences in the telencephalon of developing zebra finches. Increased expression in males was confirmed in 12 of 20 by qPCR using cDNA from the whole telencephalon; all of these appeared to be located on the Z sex chromosome. Six of the genes also showed increased expression in one or more of the song control nuclei of males at post-hatching day 25. Although the function of half of the genes is presently unknown, we have identified three as: 17-beta-hydroxysteroid dehydrogenase type IV, methylcrotonyl-CoA carboxylase, and sorting nexin 2.

**Conclusion:**

The data suggest potential influences of these genes in song learning and/or masculinization of song system morphology, both of which are occurring at this developmental stage.

## Background

**Figure 1:**
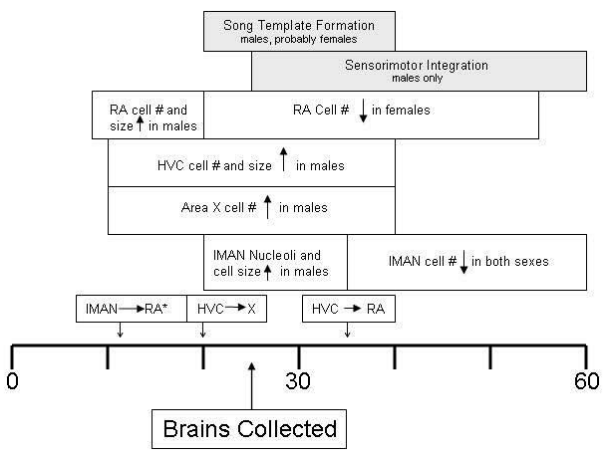
**Time-line for develoment of singing behavior and sexual differentiation of the song control nuclei in zebra finches**. *Projection apparent at this age in males. Information synthesized from [9,40,47,65,2,3].

Sexually dimorphic behaviors including types of displays, such as vocal or sexual behaviors, occur across diverse species. Particularly elegant work exists on the development of song and the brain regions associated with it in zebra finches. Adult behavior is highly dimorphic. Only males sing, and they learn songs from their fathers beginning around day 25 (Figure 1; [1-4]).

Most brain regions that control song are sexually dimorphic in adults [5]. They include the lateral magnocellular nucleus of the anterior nidopallium (lMAN), area X in the basal ganglia, the robust nucleus of the arcopallium (RA), and HVC (proper name; [6]). Area X and lMAN are important in song development, as lesions to these areas impair song learning [7]. Area X, while large in adult males, is not visible in females [8]. lMAN volume is sexually monomorphic, although as in other brain regions soma size is increased in males compared to females [9]. HVC and RA are involved in the motor production of song, and are substantially smaller in adult females than males [10].

Mechanisms regulating sexual differentiation of the song system are unclear. Administering estradiol to females early in development partially masculinizes song nuclei, and if followed by testosterone treatment in adulthood, allows females to perform rudimentary song [5]. These results have implicated steroid hormones in the masculinization of both structure and function. However, other data are inconsistent with this idea. For example, initial sexually dimorphic development of HVC appears independent of androgen and estrogen [11]. Castration of young males [12,13] and treatment with anti-estrogens [14-16] or with estrogen synthesis inhibitors [17,18] fail to inhibit masculine development. Additionally, sex differences in plasma steroid levels during development have not been conclusively identified [19-21].

Genotypic sex directly influences the relative expression of many sex-linked genes in birds; gene dosage compensation is limited [22]. The potential impact of cellular genotype on brain development was illustrated by a rare gynandromorphic zebra finch in which gonads and plumage were masculine on the right side but feminine on the left [23]. Brain cells on the right appeared to have a male (ZZ) genotype, but on the left were female (ZW), and HVC morphology was also lateralized. More recently, several genes have been discovered to exhibit increased expression in the song nuclei of juvenile males, including tyrosine kinase B [24], secretory carrier membrane protein 1 [25], estrogen coactivator L7/SPA [26], and ribosomal proteins 17 and 37 [27]. Thus, it is likely that genes and hormones, acting together within the song system, produce sex differences in singing behavior.

The goal of the present study was to identify additional genes involved in masculinization of the song system. To accomplish this, we screened developing zebra finch brains using a species-specific cDNA microarray, and followed up on 20 of those exhibiting the greatest sex difference. This male-biased expression was validated in a separate set of individuals by real-time quantitative polymerase chain reaction (qPCR). For the genes with expression that remained significantly different, *in situ *hybridization was used at day 25 post-hatching to evaluate localization in song control nuclei as in [28], to capture a period when morphological differentiation is enhanced as well as early phases of song learning.

## Methods

### Animals and Tissues

All tissue for all experiments was collected from animals living in large colony cages containing multiple males and females, along with their offspring. The day of hatching was targeted to identify genes that might influence very early stages of song system development (see Figure 1, song nuclei are not yet visible) [29]. At day 25, morphological differentiation of the neural song system is well underway [30], and both males and females are likely beginning to form templates of their fathers' songs [31]. Post-hatching day 45 is about mid-way through sensorimotor integration, a process that only occurs in males. For the microarray analysis, individual whole telencephalons (6 of each sex at each age, resulting in 48 independent samples each analyzed on a separate array) were collected following rapid decapitation from zebra finches at post-hatching days 1 (day of hatching), 25 and 45, as well as from adults (> 120 days). Brains were stored at -80°C until processing. Gonadal sex was determined using a dissecting microscope.

### Microarrays

cDNA microarrays were used following the procedures of the Songbird Neurogenomics Initiative (ESTIMA:Songbird website, ); the present study was identified as experiment #7 in the planned Community Collaborations [32]). Each array contained 20,160 spots, representing 17,214 unique genes, compiled from three zebra finch telencephalic libraries.

RNA extraction, cDNA synthesis and array hybridization using a two-color universal reference design were as described [32]. Briefly, total RNA was prepared using TRI Reagent (Ambion/Applied Biosystems, Foster City, CA). It was DNase I treated (Turbo DNase, Ambion/Applied Biosystems, Foster City, CA) and cleaned using spin columns (RNeasy, Qiagen, Valencia, CA). RNA (500 ng) was amplified using the Low RNA Input Fluorescent Linear Amplification kit (Agilent Technologies, Foster City, CA; average yield = 25 μg). The resulting aRNA was reverse transcribed using an indirect aminoallyl incorporation protocol and labeled with either Cy3 or Cy5 dyes (GE Healthcare, Piscataway, NJ). For the present study, each of the 48 samples was hybridized to a separate array, and each array was also hybridized to a common reference (labeled with the complementary Cy3 or Cy5 dye). The reference was prepared as described in [32] and included RNA from adults of both sexes. Dye labeling was balanced within groups. Slides were hybridized overnight at 42°C using SlideHyb #1 hybridization buffer (Ambion/Applied Biosystems, Foster City, CA), then washed with a series of standard saline citrate solutions, centrifuged to dry, and scanned using an Axon GenePix 4000B (Sunnyvale, CA) microarray scanner. All slide images were analyzed using GenePix Pro 6.0 software. Analyzed slide images were manually edited and aberrant spots were flagged for exclusion.

Data were analyzed as follows. The log_2 _transformation of the ratios of the loess-normalized sample:reference fluorescence intensities was used for statistical analysis. These log ratios were not corrected for background in order to provide the most stable and least variable responses in line with recent recommendations [33,34]. The log ratios were normalized for potential dye intensity bias using the loess smoothing procedure advocated by Yang et al. [35] based on the smoothing span parameter set to 0.10 for all arrays. Subsequent to loess normalization, the interquartile ranges (IQR) of the log-ratios were standardized to be of the same magnitude as the average IQR across arrays similar to that described in Yang et al. [35]. A global mixed model approach, analogous to the first stage model of Wolfinger et al[28], was used to further correct the log ratios for the global (i.e. across genes) effects of dyes and arrays. The residuals from this analysis were then corrected for array-specific print-tip and print-tip by dye effects in a series of second substage model analyses conducted separately for each array. The residuals from these analyses were used as the final normalized data for statistical inference in testing the experimental effects of interest.

The final normalized data were analyzed by a series of cDNA-specific linear models that include the effects of dye, sex, age and sex by age interaction, thereby allowing for estimated residual variances that are cDNA-specific [28]. ANOVA-based *F*-tests were then used to establish statistical significance for the overall effects of age, sex, and age by sex. The procedure of Storey and Tibsharani [36] was used to estimate the false discovery rates (FDRs) for each of these effects across genes.

### Gene Identification

The EST-derived probes on the microarray had been previously annotated by automated BLAST analysis against other genomes [32]. Clones were also re-sequenced prior to qPCR (see below). For the six targets of specific interest in the present study (below), we conducted additional manual annotation using data from the ongoing whole-genome sequencing project for the zebra finch available through the trace archives at the Washington University Genome Sequencing Center . In 3 cases we were able to confirm identifications by identifying genomic flanking sequences and constructing larger contigs which were then evaluated by BLASTn. Identities of the other three genes remain unknown; no homologues were found in the NCBI database at the time of this writing.

### Real-time qPCR

Whole telencephalons were collected from 25-day-old male and female zebra finches after rapid decapitation, and were immediately frozen on dry ice. The sex of each animal was determined by examining the gonads under a dissecting microscope. Samples were stored at -80°C until use.

RNA was isolated using methods described previously [37]. Briefly, samples were extracted with Trizol (Invitrogen, Carlsbad, CA), and DNase treated on RNeasy mini-columns (Qiagen, Valencia, CA) per manufacturer's instructions. RNA was ethanol-precipitated to increase the concentration, which was then determined by spectrophotometry. Integrity of the RNA was confirmed on 1% denaturing agarose gels.

From the arrays, 20 genes were selected for further analysis, based on a large magnitude of sexual dimorphism (males greater than females), and in some cases a known homologue or location on the Z chromosome (identified based on comparison to the chicken and zebra finch maps). All clones were re-sequenced from the 5' end on an ABI Prism 3100 Genetic Analyzer (Applied Biosystems, Foster City, CA) using T7 primers. The data were compared to those from the array (ESTIMA:Songbird website, ). Each matched its counterpart to a high degree (99–100%). As in Wade et al. [37], cDNA was simultaneously made from the individual telencephalic samples from 6 males and 7 females using the High Capacity cDNA Archive kit (Applied Biosystems, Foster City, CA) per manufacturer instructions. These samples, along with no-template controls were run in triplicate with each of 20 primer sets. In all cases, glyceraldehyde 3-phosphate dehydrogenase (GAPDH) was analyzed in parallel as a control. It was used both to document a lack of sexually dimorphic expression of a housekeeping gene and as a basis for normalization when calculating fold-differences between the sexes (ΔΔC_T_, see below). Primers were designed using Primer Express 2.0 (Applied Biosystems, Foster City, CA; Table 1). The efficiency of amplification for each set was at least 99% (determined from a simple regression of data obtained from a standard curve representing multiple RNA concentrations), and in no cases were primer dimers produced. Reactions consisted of 100 nM of each primer and cDNA equivalent to 25 ng total RNA for each individual. Power SYBR green PCR Master Mix (Applied Biosystems, Foster City, CA), was added according to manufacturer's instructions. The reactions were run on an ABI Prism PE 7000 (Applied Biosystems, Foster City, CA) using the default program.

**Table 1:** Primers Used for qPCR (All Listed 5' to 3')

GenBank Accession Number For cDNA	Forward	Reverse
CK313884	GTGTATCAAGGACCTGCCAGAAA	CGAGAGAGTGAAGGTAGTATCAACAGA

CK310754	AGGAACATTTAGGACACTGGAGTCA	GGATCTTGCATGGAGCTCTTTAGA

CK301975	CGAAGAGTCCATACTGAAATAAACAACA	TTCCATGTGCAAAATTCAGATGA

CK301827	AGTTCATCCGAAACCTACCATCAT	GCTTAAGAGTGGCCCCTTTCTAT

DV950099	GCGCCTGGAGCAACTTGAT	TTCCATTCTGCCTCCTGCTT

DV945668	ATCCTCACTTACTGCCCAGGTAGA	CTTCAGCTTCTTCTTGCCTTGTATT

CK303566	GAGCAATGTAGGTAATGTGGGTCAT	TTTGGCAGCAATCATCAA

CK303668	GCTGATTCCCCGAGAAACCT	CAGGCAGCGATGCTCTGTT

CK303992	TTTTCTTTGCACATTTTAGCTGAATAA	TTAACCTAAAGTCTAATCACACCAA

DV952571	CAGCTGGGCTGAACATTTGAT	ACAACTCTCTACCCATGTGTGGAA

CK306648	TCTTCGGGAGGCCGTTCTA	TGTCATAAGCATGTCACGTTCTTGT

DV956689	GTCCCCTGCCTATCTTCCTTTT	GGGATTTTGAATGAGCCCTTT

CK303187	GTTGCCTATTCTGTGGCCTGTT	GATTCCCACACTGAAAGCAGAGA

CK306803	GCAGTAAAAGGTGTGTTTGACCAT	CTTGCCACTTCTGCCAGCAT

CK308336	GGGCCATCACCTACTACCTGAA	CACCGTGCGGCGATTT

CK308959	TGGTTGTTGCTTGACATTTGAAA	CTACATGGCAGAGATAACGATTTGA

DV946640	CAGCAAATTAGCAACAAAATACAT	GCTGGAGGCACAATACAACCA

CK310795	GGAGGTTCGAAGAGGAAGGAA	TCCCATAATCTTGCACTGGAATAA

DV947064	TGTTTGTTTCAGTGTTTCCTTGTGT	GACTTGTGGATAACCTTACAGACATTTT

DV948036	CATGGAGTAATGCACACCAGTCTAT	CGGAGTAAAGAGCTGTTCATCAA

The triplicate C_T_s (threshold cycles) from each individual were averaged, and sex differences were evaluated by two-tailed t-tests. Bonferroni corrections were used (α = 0.05/20 = 0.0025). Average ratios of male to female expression were calculated using ΔΔC_T_[25,37-39].

### In Situ Hybridization

Colonies used to generate probes were obtained from glycerol stocks, and plasma DNA was isolated using Wizard Plus Minipreps (Promega, Madison, WI). Clones were re-sequenced as described above. To obtain T3 (anti-sense) & T7 (sense) probes we used Qiagen Maxi Prep kit (Valencia, CA), and linearized the templates using XhoI (T3) and NotI (T7). In all cases, T3 was the anti-sense strand and T7 was the sense strand. Cold transcription reactions were performed to confirm product quality and the correct size.

Six birds of each sex were rapidly decapitated at post-hatching day 25. Whole brains were frozen in cold methyl-butane and stored at -80°C. They were coronally sectioned (20 μm) and mounted onto SuperFrost Plus slides (Fisher Scientific, Hampton, NH). Six series of sections representing the whole telencephalon were collected, and stored at -80°C with desiccant.

*In situ *hybridization for each mRNA was conducted as in [37] and [25]. Two adjacent sets of tissue sections from each animal (one for antisense and one for sense probes) were warmed to room temperature, rinsed in phosphate buffered saline (PBS), fixed in 4% paraformaldehyde, and washed in 0.1% diethylpyrocarbonate-treated (DEPC) water, followed by PBS. Slides were incubated for 10 minutes in 0.25% acetic anhydride in 0.1 M triethanolamine, rinsed in PBS, and dehydrated in ethanols, and air dried for 10 minutes. They were pre-hybridized in a solution containing 1× hybridization buffer (2.5 M NaCl, 1 M Tris, 0.5 M EDTA, 1 M DTT, 1× Denhardts, 1 mg/ml yeast tRNA, 50% dextran sulfate, and DEPC-H_2_O) and 50% formamide at 55°C for 2 hours. Slides were then hybridized overnight at 55°C with 200 μl of 1× hybridization buffer, 10% dextran-sulfate, 50% formamide, and 5 × 10^6 ^cpm ^33^P-UTP-labeled RNA probe (antisense or sense). These probes were prepared using the MAXIscript In Vitro Transcription Kit with T3/T7 RNA polymerases (Ambion, Austin, TX). The next day, slides were removed from hybridization buffer and washed sequentially in 4 × SSC and 2 × SSC at 55°C and room temperature, followed by an incubation in 2 × SSC with RNase A (20 μg/ml) at 37°C for 30 minutes with slow agitation. Slides were then rinsed in 2 × SSC at 37°C for 15 minutes and briefly in 0.1 × SSC at 60°C. The tissue was dehydrated in ethanols with 0.3 M ammonium acetate. Slides were air dried and exposed to Hyperfilm MP (Amersham Biosciences, Piscataway, NJ) with an intensifying screen (BioMax Transcreen LE; Eastman Kodak, Rochester, NY) at -80°C for four days to confirm signal and check for enhanced expression in song system nuclei, and were then dipped in NTB emulsion (Eastman Kodak, Rochester, NY) and incubated at 4°C for three to six weeks depending on the abundance of mRNA. They were then developed using Kodak Professional D-19 Developer and Fixer (Eastman Kodak, Rochester, NY) and lightly counter-stained with cresyl violet.

For the probes demonstrating specific labeling in one or more of the song nuclei (far greater in sections exposed to anti-sense compared to sense, and elevated compared to surrounding regions of the telencephalon), the density of silver grains was assessed as follows. Without knowledge of the sex of the birds, each brain area of interest was first located using brightfield microscopy (landmarks identified using the cresyl violet nissl stain). The density of labeling was quantified in 6 animals of each sex (except for the gene associated with GenBank DV956689, for which, due to a processing problem, one female was excluded from analysis) and on both sides of the brain in all sections in which each region of interest was readily identifiable (a total of 4–8 per region per bird). The area covered by silver grains within a 264 μm × 198 μm box was determined using density slice function in NIH (Scion) Image on darkfield images. In lMAN, HVC and RA, this box covered approximately 95% of the total area in females and roughly 50% of the HVC and RA of males as they are larger; the overall size of lMAN is similar in the two sexes. The box also covered about 50% of area X in males (boxes corresponding to the same portion of the medial striatum were used in females, in which area X is not visible). For each anti-sense treated section, an adjacent sense section was quantified. The control, sense, values were then subtracted from the corresponding antisense values, and the resulting specific densities were averaged across sections within individuals. A t-test was conducted for each brain region for each gene, and the alpha-level was set based on the number of brain regions measured for each gene (Bonferroni correction).

### Southern Blot Analyses

For genes with enhanced mRNA expression in male song nuclei, we verified their location on the Z chromosome by documenting significantly increased labeling in males versus females on genomic Southern blots. The location was later confirmed using BLASTn to compare our sequences to the 2008 release of the zebra finch genome . For the Southerns, whole blood was collected from adult individuals of both sexes in heparinized capillary tubes after alar venipuncture, and stored at -20°C. Genomic DNA was isolated using Qiagen DNeasy Blood and Tissue Isolation kit (Valencia, CA) following manufacturer's instructions (for blood with nucleated erythrocytes). Concentration was determined by spectrophotometer. DNA (10 μg) was then digested overnight at 37°C with HindIII, or, in one case, EcorI (due to internal cut sites; New England Biolabs, Ipswich, MA).

Digested DNA from 8 adult males and 8 females was loaded onto a 0.9% agarose gel, with samples from each sex in alternate lanes, and run at approximately 40 volts for 2 days, or until the bromphenol blue dye front was at the bottom of the gel. It was depurinated for 10 minutes in 0.2 N HCl, rinsed, denatured for 15 minutes in 15 M NaCl and 0.5 M sodium hydroxide, and rinsed again. The gel was neutralized for 30 minutes in 1.5 M NaCl in 0.5 M Tris. After a final rinse, it was soaked in 20 × SSC. The DNA was transferred to a Hybond-N+ membrane (GE Healthcare, Piscataway, NJ) overnight, and fixed using a UV stratalinker (Stratagene, La Jolla, CA). Probing for the six genes of interest was conducted across three replicate membranes. After stripping the membranes, they were then re-probed for GAPDH as a control (at 68°C; cloned using primers generated from the zebra finch sequence, GenBank: AF255390; in pBluescript). After a 2 minute wash in 5 × SSC buffer, the membrane was prehybridized at 68°C for at least one hour in 5 × SSC, 5 × Denahrdts, 0.5% SDS, and 5 mg of denatured herring sperm DNA (Sigma, St. Louis, MO). The membrane was then hybridized for 2 days at 68°C in 5 × SSC, 5 × Denhardts, 0.5% SDS, 2 ng denatured DNA, and 40 × 10^6 ^cpm probe.

Probes were individually prepared using 5 ng of template that was amplified using 2.5 units of Platinum Taq High Fidelity (Invitrogen, Carlsbad, CA) with 2 mM Magnesium sulfate, 0.2 mM dNTPs (all except dCTP), 2 μM dCTP, 9 μM T3 & T7 primers, and 5 μCi ^32^p-dCTP.

Following warming at 94°C for 5 minutes, reactions were subjected to 33 cycles of: 94°C for 30 seconds, 55°C for 30 s, and 68°C for 1 to 1.5 minutes (depending on the size of the product), with a final extension at 68°C for 10 minutes. Probes were purified using a spin column containing G50 sephadex beads (Sigma, St. Louis, MO) in NETS. Membranes were washed at 68°C in 2 × SSC with 0.1% SDS twice for 5 minutes, and in 1 × SSC with 0.1% SDS twice for 10 minutes prior to exposure to Hyperfilm MP (Amersham Biosciences, Piscataway, NJ) at least overnight at -80°C, depending on the strength of the signal.

The mean optical density of the band of interest for each individual was quantified using NIH (Scion) Image. This value was divided by the value for the corresponding GAPDH band. The resulting data were analyzed using t-tests with (α = 0.008, 0.05/6).

## Results

### Microarrays

We detected 2419 spots on the array that showed significant effects of sex (FDR p < 0.05), half of them (49%) with increased expression in males compared to females. However, of the targets with average effect sizes of 1.5-fold or greater, 300 were increased in males and 51 increased in females. A very large number (16,497) of the cDNAs exhibited a significant effect of age. This result is not particularly surprising, as the four ages investigated span the entire period of maturation for these birds, thus countless structural and functional changes in the brain would be expected. A relatively small number of cDNAs expressed a significant sex × age interaction, just 114 of the more than 20,000 spots on the array. These will be pursued in the future.

As our present goal was to identify genes involved in any aspect of masculinization of the song system, we chose to follow up on a set of 20 with increased expression in males compared to females across all ages. Most of these genes were initially selected based on substantially greater expression in males compared to females and location of the orthologous sequence in chicken on the Z chromosome (which was the only information available for birds at the time). In a few cases, genes were chosen for further analysis due to a high degree of sexual dimorphism in the absence of additional information. Because some gene identities have not been established (see above), we use GenBank accession numbers here to label genes.

### qPCR

Of the 20 cDNAs chosen for further analysis, 12 showed male-biased expression via qPCR (Table 2). Two of these, with GenBank accession numbers DV946640 and CK306803, represented cDNAs from the same gene, sorting nexin 2 (SNX 2). Therefore, DV946640 was excluded from further analyses. GAPDH expression never differed significantly between the sexes.

**Table 2:** Sexually Dimorphic Expression in Day 25 Zebra Finches Detected with cDNA Microarrays and Real-time qPCR

GenBank Accession Number	Z or W*	Tentative Identification**	Male/Female Ratio on Array (across ages)	Array pFDR	Male/Female Ratio using qPCR	qPCR t, p-values
CK313884	Z	17-beta-hydroxysteroid dehydrogenase type IV	2.05	.023	2.37	7.09, 0.0001

CK310795	Z	Methylcrotonyl-CoA carboxylase beta chain	1.20	<.0001	2.25	5.30, 0.0003

CK303566	Z	Unknown	2.03	<.0001	2.05	5.95, <0.0001

CK310754	Z	Aprataxin/forkhead-assoc.domain	2.06	.0002	2.53	4.65, 0.0008

DV956689	Z	Unknown	2.00	.0013	1.89	5.44, 0.0002

CK308959	Z	Unknown	2.20	<.0001	2.8	3.79, 0.0022

CK306648	Z	Unknown	1.99	<.0001	2.18	5.54, 0.0002

CK303187	Z	KIAA 1797 (large protein family)	2.43	<.0001	1.96	4.44, 0.0012

CK306803	Z	Sorting Nexin 2	2.19	.0015	2.19	6.97, 0.0001

DV946640***	Z	Sorting Nexin 2	2.10	.0004	2.07	5.11, 0.0003

DV947064	Z	Unknown	1.98	.006	2.32	4.33, 0.0012

DV948036	Z	Unknown	2.00	.015	2.29	4.91, 0.0005

*Location on Z or W chromosome

**Based on August 6, 2008 zebra finch genome release (not yet annotated).

***Later determined to represent the same gene as CK306803, so excluded from further analysis

### In situ Hybridization

Of the 11 cDNAs carried forward in the analysis, expression of 8 was detected in one or more song nuclei. Labeling was also detected in restricted regions outside of the song system, but was not quantified in the present study. The remaining 3 [GenBank: CK310754, DV947064, and DV948036] showed no specific staining in song control regions; they will not be discussed further.

**Figure 2:**
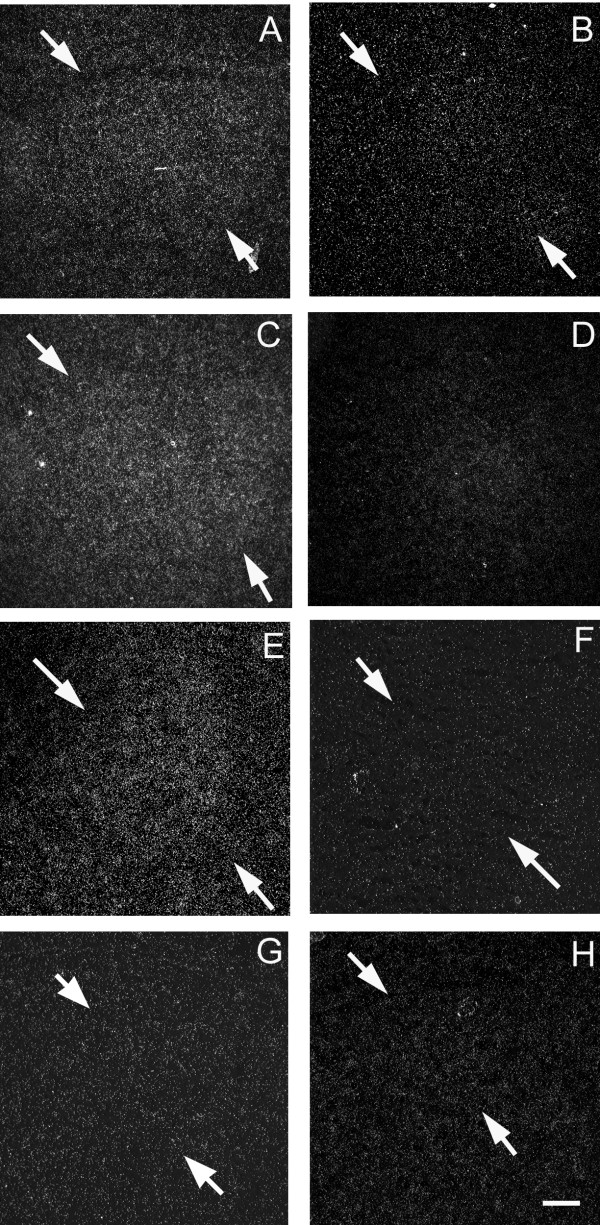
**Darkfield images from *in situ *hybridization depicting sexually dimorphic mRNA expression for **CK303566**in the zebra finch song system at day 25 post-hatching**. Arrows delineate borders of song regions. This gene showed the most extensive sex differences in expression. In lMAN (lateral magnocellular nucleus of the anterior nidopallium) males (A) show higher levels of mRNA expression (i.e. higher densities of silver grains) than do females (B). Similar differences were obtained in the portion of the medial striatum which contains area X in males (C), although area X is not morphologically distinct in females (D), and in RA (robust nucleus of the arcopallium, panels E = males, F = females). However, in HVC, no specific labeling was detected in either sex (panel G = male, panel H = female). Scale bar = 200 μm for lMAN and area X; 100 μm for HVC and RA.

Specific expression of CK303566 was detected in lMAN, Area X and RA, but not HVC (Figure 2). The density of labeling was greater in males than females in all three areas (lMAN: t = 3.32, p = 0.008; Area X: t = 3.35, p = 0.007; RA: t = 3.89, p = 0.003; Table 3).

**Table 3:** Summary of Sexually Dimorphic Expression of Eight Putative Genes at Post-hatching Day 25

GenBank Accession Number	Tentative Identification	lMAN	Area X	HVC	RA
CK313884	17β-HSD4†	NS*	NS	**M>F** **3.56**^a^	--

CK310795	Methylcrotonyl-CoA	**M>F** **2.30**	**M>F** **3.90**	--	--

CK303566	Unknown	**M>F** **2.68**	**M>F** **2.48**	--	**M>F** **11.81**

DV956689	Unknown	**M>F** **4.65**	**M>F** **3.96**	--	--

CK308959	Unknown	NS	--	**M>F** **3.80**	--

CK306648	Unknown	NS	--	--	--

CK303187	KIAA 1797	NS	--	--	--

CK306803	SNX2†	NS	**M>F** **3.20**	**M>F** **7.41**	--

*Area measured, but no significant difference between males and females were found.

__ Area did not show specific staining within song nuclei on film or on slides, and therefore was not quantified.

†17β-HSD4 = 17-beta-hydroxysteroid dehydrogenase type IV; SNX2 = Sorting Nexin 2

^a^Magnitude of sex difference (Male mean/female mean)

**Figure 3:**
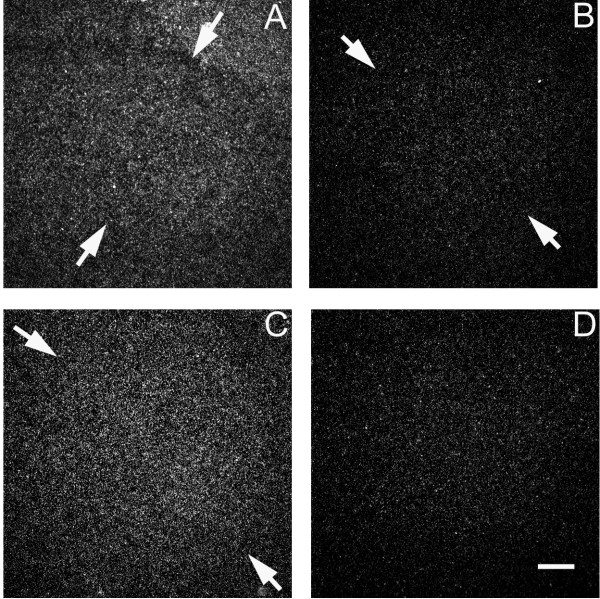
**Darkfield images from *in situ *hybridization depicting sexually dimorphic mRNA expression for **CK310795**(Methyl-crotonyl carboxylase CoA) in the zebra finch song system at day 25 post-hatching**. Arrows delineate borders of song regions. In lMAN (lateral magnocellular nucleus of the anterior nidopallium), males (A) show higher levels of mRNA expression (i.e. higher densities of silver grains) than do females (B). Expression was also increased in males (C) compared to females (D) in the portion of the medial striatum that contains area X in males. Scale bar = 200 μm.

CK310795 (Methylcrotonyl-CoA carboxylase beta chain) showed specific expression in two song nuclei – area X and lMAN. In both cases, it was increased in males compared to females (Area X: t = 3.56, p = 0.005; lMAN: t = 2.92, p = 0.015; Figure 3).

DV956689 was also expressed in area X and lMAN, but not in HVC or RA. Males showed significantly higher levels of expression than did females in both areas (area X: t = 3.85, p = 0.004; lMAN: t = 3.86, p = 0.004).

**Figure 4:**
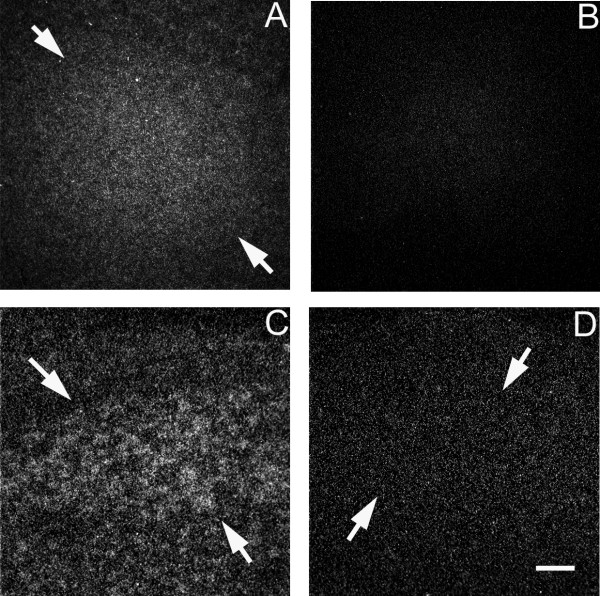
**Darkfield images from *in situ *hybridization depicting sexually dimorphic mRNA expression for **CK306803**(Sorting nexin 2) in the zebra finch song system at day 25 post-hatching**. Arrows delineate borders of song regions. In area X (or the portion of the medial striatum containing it), males (A) showed higher levels of mRNA expression than did females (B). The increased expression of this gene in males (C) compared to females (D) was also detected in HVC. Scale bar = 200 μm for area X and 100 μm for HVC.

Three of the four song nuclei (lMAN, Area X, and HVC) exhibited specific labeling indicating SNX 2 mRNA [GenBank: CK306803]. It was increased in males in area X and HVC (t = 2.85, p = 0.017; t = 11.10, p < 0.001, respectively; Figure 4). Expression between the sexes, however, did not differ in lMAN (t = 0.94, p = 0.370).

**Figure 5:**
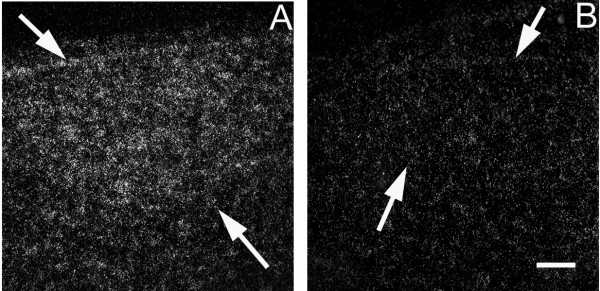
**Darkfield images from *in situ *hybridization depicting sexually dimorphic mRNA expression for **CK313884**(17-beta-hydroxysteroid dehydrogenase type IV) in HVC at day 25 post-hatching**. Arrows delineate borders of song region. Males (A) showed higher levels of mRNA expression than did females (B). Scale bar = 100 μm.

CK313884 (17-beta-hydroxysteroid dehydrogenase type IV) showed enhanced expression compared to surrounding tissue in lMAN, area X, and HVC, but not in RA. Males had higher levels of expression in HVC than did females (t = 3.13, p = 0.008; Figure 5). The sexes did not differ in the other two areas (lMAN: t = 1.63, p = 0.135; area X: t = 1.55, p = 0.150).

CK308959 mRNA appeared specific to lMAN and HVC. In HVC, it was increased in males compared to females (t = 3.38, p = 0.007). Males also showed somewhat higher levels of expression than did females in lMAN, though this difference was not statistically significant (t = 2.25, p = 0.048).

Only one area, lMAN, showed specific labeling for CK306648, with the intensity substantially greater than in surrounding tissue. It, however, did not significantly differ between the sexes (t = 0.51, p = 0.619). Results for CK303187 were the same (t = 0.47, p = 0.651).

### Southern Blot Analyses

The corrected optical density representing each of the six genes with sexually dimorphic expression in the song system was significantly higher in males compared to females (all t > 3.464, all p < 0.004; data not shown). In parallel, each of the sequences represented in Table 2 shared substantial identity with portions of the zebra finch Z-chromosome (2008 map, not yet annotated).

## Discussion

We found robust sex differences in gene expression in the developing zebra finch brain, and identified six genes that are both sex-linked and differentially expressed in the male compared to female song system. This is the period when song memories are first forming and morphological differentiation is occurring at a rapid rate [29,30,40].

All six of these genes map to portions of the Z chromosome in zebra finches (as well as chicken), and our Southern blot analysis confirmed they are Z-linked in zebra finches. Most Z-chromosome mRNAs in birds exhibit increased expression in male compared to female birds, as dosage compensation is limited [22,41,42], unlike the analogous situation in mammals where dosage compensation prevents enhanced expression in females of most X-linked genes. We anticipate that a number of the ~2400 sex-biased genes we detected in our microarray study will map to sex chromosomes. However, local dosage compensation does exist in birds and varies across ontogeny and tissues, which suggests some active regulation associated with key functions [43].

Out of the eleven genes we initially chose for analysis by *in situ *hybridization, three [GenBank: CK310754, DV947064, DV948036] showed sexually dimorphic expression via qPCR on RNA/cDNA from the whole telencephalon, but the mRNA was neither confined to nor enhanced in song nuclei. Of the eight remaining, two [GenBank: CK306648, CK303187] appeared to show specific expression in lMAN, but significant sex differences were not detected via *in situ *hybridization. The results from these five genes thus might reflect either a generalized increase in males, or stem from specific, functional increases in regions of males outside of the song control system.

The other six genes appear important for the song system specifically, since expression was higher in song system nuclei relative to surrounding tissue. Importantly, sexually dimorphic expression of each of these genes is localized to particular brain areas. For example, while some mRNA was detected outside of the song nuclei, expression of all six was clearly enhanced in lMAN compared to neighboring tissue, yet significant sex differences within lMAN existed for only half of them. Similarly, five of the genes showed increased expression in Area X compared to the surrounding tissue, and of these, four exhibited significantly greater expression in males than females while one did not. If one considers the six individual genes across the four song nuclei investigated, not one of them showed sexually dimorphic expression in all of the areas (see Table 3). These indications of specificity imply restricted functions, and the pattern of expression provides some clues as to the details regarding mechanism.

### LMAN

LMAN is involved in song learning. Lesions early in development disrupt normal learning in males but in adulthood do not alter singing behavior [44,45]. LMAN volume is relatively large at day 25 in both sexes, and begins rapidly regressing thereafter [46]. Sex differences are apparent in cellular structure of lMAN, even though the region is volumetrically monomorphic: nucleoli and neuronal soma sizes are larger in males relative to females [9]. In males, lMAN projects to RA early (day 15), and this connection reorganizes (lMAN projections to RA regress, and connections from HVC begin to form at these same synapses) around day 35 [40]. The regression of terminals from lMAN is correlated with the entrance of axons into RA from HVC [47]. Increased expression of the synelfin (alpha-synuclein) gene has been detected in male lMAN during this developmental period [48] with subsequent changes in the presynaptic protein in terminals onto RA [49].

Three genes showed significantly enhanced expression in the lMAN of males relative to females at post-hatching day 25 [GenBank: CK310795, CK303566, DV956689]. CK310795 has been identified as Methylcrotonyl-CoA carboxylase beta chain, and is involved in the catabolism of leucine, important for releasing energy in the brain [50]. Increased leucine catabolism is marked during operant conditioning training in mice [51], and humans deficient in 3-methylcrotonyl-CoA carboxylase can exhibit motor deficits, learning disabilities, attention-deficit disorders, as well as a reduction in white matter [52,53]. These findings suggest that CK310795, and possibly the other two genes, may facilitate song learning. They might interact with NMDA receptors, as MK-801 (receptor antagonist) binding was higher in lMAN at day 30 than in adults, and injections given to juvenile males between day 21 and 50 impaired song learning [54].

### Area X

Area X is involved in learning patterns of motor production for song [55,56]. Lesions of area X during song learning disrupt vocal output and prevent song crystallization [45,57]. At this age, area X in males is approximately half the adult volume, but rapidly increases with recruitment of new neurons [29]. This area never develops in females [29]. HVC projects to area X by day 20 in males [47].

Four mRNAs investigated in the present study were enhanced in area X of males relative to females, and three of these exhibited the same pattern in lMAN [GenBank: CK310795, CK303566, DV956689]. Since area X and lMAN are both involved in song learning, it is reasonable to hypothesize that these genes play some role in this process.

CK306803, identified as sorting nexin 2 (SNX2), showed enhanced expression in area X of males. In mammals, SNX2 is a retromer component (which mediates the retrieval of transmembrane receptors) that is important during development, especially in the degradation of chemicals, including toxins [58]; the deletion of SNX2 and structurally similar SNX1 simultaneously in mice embryos has lethal consequences [59]. Depletion in humans of other, related, retromer subunits, Vps26 and Vps35, which require either SNX1 or SNX2 in order to associate with endosomes, increases amyloid-β peptide production associated with Alzheimer's disease [60]. Since the formation of amyloid plaques is implicated in memory deficits, SNX2 might be involved in maintaining neural circuitry that is essential for learning in males.

### HVC

HVC is involved in motor output of song and is thought to be the primary entry of auditory information into the song pathway [61]. It has connections to both motor learning and output nuclei; one that projects to area X develops at day 20, and one to RA around day 35. These projections are absent in females [47]. Although HVC is already sexually dimorphic in volume and cell number at day 25, new neurons are recruited in males until day 30, and this region does not fully mature until day 60 (reviewed in [9]). Cell death begins around day 30 in females, and leads to further differentiation of the region [9].

Three mRNAs exhibited enhanced expression in males relative to females [GenBank: CK313884, CK308959, CK306803] in HVC, including SNX2 [GenBank: CK306803]. Given that HVC and Area X, which showed a similar sex difference, are both incorporating cells during this period, SNX2 may promote the survival or incorporation of new cells.

Two genes, CK313884 and CK308959, showed sexually dimorphic expression in only one brain area, HVC. At day 25, this area is unique in that it is the only song region that contains substantial levels of estrogen receptors [62]. Additionally, the closing of the song template formation phase (which occurs around day 40) is marked by a decrease in estrogen receptor-immunoreactive cells activity in male HVC [62]. CK313884 might play a role in the decreased estrogen receptor activity, as it converts estradiol into estrone, for which estrogen receptors have a lower affinity [63]. CK308959 may serve similar functions, promoting cell survival in HVC, or preventing cell death. CK308959 may also interact with androgen receptors, since androgen receptor binding increases markedly beginning at day 25 in both HVC and lMAN [40].

### RA

RA projects to the respiratory tract and syrinx necessary for producing song [64]. This song nucleus receives inputs from two other song regions: lMAN and HVC [47]. Sexual differentiation of RA morphology occurs earlier than other song nuclei (around day 6); volumetric differences between the sexes are largely due to cell death in females [65]. However, synaptic densities and neuron soma sizes increase in males [4].

CK303566 showed enhanced expression in the RA of males. It was also increased in males in lMAN, an area that also has strong connections with RA early in development. Since lMAN likely affects the synaptic rearrangement in RA, this gene may play a role in the shift in connectivity (from lMAN to HVC) that occurs at this age.

## Conclusion

The present study identified more than 2400 putative genes with sex differences in expression across multiple ages in zebra finches; of a subset analyzed with qPCR, 12 mRNAs (2 of them representing the same gene) with substantially greater expression in males were validated using qPCR. Sex differences in mRNA expression for six were localized to one or more song control regions. The patterns of gene expression were not uniform across the song system, which allowed the formulation of some hypotheses regarding the function of these genes. In particular, we have identified genes that may facilitate cell incorporation and cell survival, or that may enhance or maintain learning and memory circuitry. As all are located on the Z-chromosome, they have the potential to be early players in the cascade of events causing masculinization of the structure and/or function of the song system. Future studies will focus on the mechanisms through which that occurs, including whether and how they may alter the way the brain responds to steroid hormones in the sexual differentiation process.

## Authors' contributions

The arrays were designed and hybridized as part of the Songbird Neurogenomics Initiative , which included DC, KR and JW, among others. MT and JW designed other portions of the study. MT carried out the *in situ *hybridizations, performed statistical analyses on qPCR, *in situ *hybridizations, and Southerns, and drafted the manuscript, which JW also worked on. CP performed qPCR and Southerns. KR carried out the microarray hybridizations, RT performed the statistical analyses on the microarray data. All authors read and approved the final manuscript.
